# Unraveling the role of lipid droplets and perilipin 2 in bovine luteal cells

**DOI:** 10.1096/fj.202400260RR

**Published:** 2024-06-15

**Authors:** Michele R. Plewes, Heather A. Talbott, Micah B. Schott, Jennifer R. Wood, Andrea S. Cupp, John S. Davis

**Affiliations:** 1 Olson Center for Women’s Health, Department of Obstetrics and Gynecology, University of Nebraska Medical Center, Omaha, Nebraska, USA; 2 Veterans Affairs Nebraska Western Iowa Health Care System, Omaha, Nebraska, USA; 3 Department of Biochemistry and Molecular Biology, University of Nebraska Medical Center, Omaha, Nebraska, USA; 4 Department of Animal Sciences, University of Nebraska–Lincoln, Lincoln, Nebraska, USA

**Keywords:** corpus luteum, lipid droplets, perilipin 2, progesterone, prostaglandin F2α, steroidogenesis

## Abstract

Steroidogenic tissues contain cytosolic lipid droplets that are important for steroidogenesis. Perilipin 2 (PLIN2), a structural coat protein located on the surface of lipid droplets in mammalian cells, plays a crucial role in regulating lipid droplet formation and contributing to various cellular processes such as lipid storage and energy homeostasis. Herein, we examine the role that PLIN2 plays in regulating progesterone synthesis in the bovine corpus luteum. Utilizing gene array databases and Western blotting, we have delineated the expression pattern of PLIN2 throughout the follicular to luteal transition. Our findings reveal the presence of PLIN2 in both ovarian follicular and steroidogenic luteal cells, demonstrating an increase in its levels as follicular cells transition into the luteal phase. Moreover, the depletion of PLIN2 via siRNA enhanced progesterone production in small luteal cells, whereas adenovirus-mediated overexpression of both PLIN2 and Perilipin 3 (PLIN3) induced an increase in cytosolic lipid droplet accumulation and decreased hormone-induced progesterone synthesis in these cells. Lastly, in vivo administration of the luteolytic hormone prostaglandin F2α resulted in an upregulation of PLIN2 mRNA and protein expression, accompanied by a decline in serum progesterone. Our findings highlight the pivotal role of PLIN2 in regulating progesterone synthesis in the bovine corpus luteum, as supported by its dynamic expression pattern during the follicular to luteal transition and its responsiveness to luteotropic and luteolytic hormones. We suggest PLIN2 as a potential therapeutic target for modulating luteal function.

## 1 | INTRODUCTION

Lipid droplets are dynamic organelles central to lipid metabolism and energy homeostasis that have recently gained recognition for their pivotal role in ovarian physiology.^1–3^ These organelles store neutral lipids and are encapsulated by a phospholipid monolayer embedded with lipid droplet-associated proteins, notably perilipins (PLIN1–5). These proteins are crucial for stabilizing the lipid droplet structure and facilitating protein complex assembly on the surface.^4^ In the corpus luteum, PLIN2 (also known as adipose differentiation-related protein, ADRP) is notably expressed in steroidogenic luteal cells.^5^ This structural coat protein is implicated in regulating fatty acid mobilization and lipid droplet formation across various mammalian cells^6^ and plays a key role in ovarian processes such as follicular maturation,^7^ oocyte development,^8^ angiogenesis,^9^ and steroidogenesis.^2^ Although lipid droplets are ubiquitous across tissues and have been extensively studied in adipocytes, those in luteal cells exhibit a distinctive composition, being enriched not only with triglycerides but also with cholesteryl esters. This unique composition underscores their vital role in both energy provision and as substrates for progesterone synthesis.^5,10^

The ovary is a dynamic organ that undergoes remarkable structural and functional changes.^11^ The functional unit of the ovary is the ovarian follicle, which comprises oocytes, theca cells, and granulosa cells. Post-ovulation, these follicles experience significant transformations, a process initiated by luteinizing hormone (LH), which is synthesized and secreted by the anterior pituitary gland. LH induces follicle rupture to release the ovum and stimulates the transition of theca and granulosa cells from follicular to luteal, forming small and large luteal cells in the corpus luteum.^12^ Subsequently, LH stimulates progesterone production in luteal cells through the cAMP/Protein Kinase A (PKA) pathway,^10,13^ a crucial process for establishing and maintaining pregnancy. Previous studies indicate that LH regulates pathways that promote the activation of hormone-sensitive lipase (HSL, also known as LIPE) and the mobilization of cholesterol, a process that is required for progesterone synthesis.^2,10,14^ In this process, lipid droplets play a crucial role.^2^ Both small and large luteal cells react to LH stimulation; however, small luteal cells show a notably higher responsiveness to LH and to activators of the cAMP/PKA signaling pathway compared to large luteal cells.^6^ Furthermore, under basal conditions, large luteal cells have a higher capacity to synthesize progesterone compared to small luteal cells.^15^

Luteolysis is a natural developmental process essential for regulating the female reproductive cycle.^16^ At the onset of luteolysis, there is a precipitous decline in serum progesterone concentrations, followed by the structural demise of the gland.^17^ Uterine-derived Prostaglandin F2α (PGF2α) initiates luteolysis in a variety of species, including domestic animals,^18–21^ rodents,^22,23^ guinea pigs,^24^ rabbits,^25^ and primates, with both endogenous PGF2α and estrogen playing roles in primates.^26^ PGF2α exerts its effects via the phospholipase C-intracellular calcium-protein kinase C (PKC) pathway. In the ovine, luteal regression is marked by a sudden increase in free cholesterol and triglyceride levels and a notable alteration in fatty acid composition.^27^ Pregnancy, on the other hand, was reported to inhibit or reverse these changes.^27^ While PGF2α is well known to be involved in the regression of the CL, its role in lipid synthesis and other processes within the corpus luteum remains unclear.

Given the unique enrichment of lipid droplets in ovarian luteal cells,^2,5,28–30^ our study aims to explore the role of PLIN2 in regulating lipid metabolism and steroidogenesis in the corpus luteum. We characterize PLIN2 expression in follicular and luteal cells and examined changes during in vitro differentiation of theca and granulosa cells. Employing genetic knockdown and overexpression techniques in small luteal cells, we investigate PLIN2’s impacts on lipid droplet abundance and progesterone synthesis. Additionally, we assess PLIN2 expression in vivo following administration of a luteolytic dose of PGF2α. This comprehensive approach provides insights into the regulation, function, and significance of lipid droplets in follicular and luteal cells, shedding light on the complex interplay between these organelles and essential processes in the corpus luteum.

## 2 | MATERIALS AND METHODS

### 2.1 | Reagents

Penicillin G-sodium, streptomycin sulfate, HEPES, bovine serum albumin (BSA), deoxyribonuclease l, fetal bovine serum (FBS), Tris–HCl, sodium chloride, ethylenediaminetetraacetic acid (*EDTA*), ethylene *glycol*-bis(β-aminoethyl ether)-*N*,*N*,*N*′,*N*′-*tetraacetic* acid (EGTA), sodium fluoride, Na_4_O_2_O_7_, Na_3_VO_4_, Triton X-100, glycerol, dodecyl sodium sulfate, β-mercaptoethanol, bromophenol blue, Tween-20, paraformaldehyde, and phorbol 12-myristate 13-acetate (PMA) were purchased from Sigma-Aldrich (St. Louis, MO, USA). The phosphate buffer solution, DMEM (calcium-free, 4.0 g/L glucose), Penicillin Streptomycin Solution, trypan blue, Halt Protease, and Phosphatase Inhibitor Cocktail were purchased from Invitrogen Corporation (Thermo Fisher, Carlsbad, CA, USA). The opti-MEM, M199 culture medium, insulintransferrin-Selenium (100×), and gentamicin sulfate were purchased from Gibco (Thermo Fisher, Waltham, MA, USA). Collagenase was purchased from Atlanta Biologicals (Flowery Branch, GA, USA). Bovine LH was purchased from Tucker Endocrine Research Institute (Atlanta, GA, USA). Nitrocellulose membrane, Lipofectamine RNAiMAX Transfection Reagent, No. 1 glass coverslips, microscope slide, and chemiluminescent substrate (SuperSignal West Femto) were from Thermo Fisher Scientific (Waltham, MA, USA). Fluoromount-G and clear nail polish were purchased from Electron Microscopy Sciences (Hastfield, PA, USA). Forskolin was purchased from EMD Millipore (Burlington, MA, USA). BCA protein assay and 4–20% Mini-PROTEAN^®^ TGX^™^ precast protein gels were purchased from Bio-Rad (Hercules, CA, USA) and the non-fat milk was from a local Kroger (Cincinnati, OH, USA). Lutalyse^®^ was purchased from Zoetis Inc. (Kalamazoo Michigan, MI, USA). The *ImmPACT DAB Peroxidase (HRP) Substrate Kit and Antigen unmasking solutions* were purchased from *Vector Laboratories* (Newark, CA, USA). An enzyme-linked immunosorbent assay (ELISA) kit for progesterone was purchased from DRG International, Inc. (Springfield, NJ, USA). The ImmuChemTM Coated Tube Progesterone 125I radioimmunoassay (RIA) kit was purchased from ICN Pharmaceuticals, Inc. (Costa Mesa, CA, USA). The siRNA, siControl, and siPLIN2 [ON-TARGETplus PLIN2 (J-019204–10-0005) SMARTpool, 85% matched to bovine] were purchased from Dharmacon (Lafayette, CO, USA). The adeno (Ad) viruses Ad.PLIN2 (VH811755) and Ad.PLIN3 (VH802938) were purchased from Vigene Biosciences, Inc. (Rockville, MD, USA). Table 1 lists all the antibodies used in the study.

### 2.2 | Follicular cell isolation

All procedures were approved by the Animal Care and Use Committee at the University of Nebraska-Lincoln. The University of Nebraska-Lincoln is AAA-LAC-certified. Non-lactating, composite beef cows [25% MARC III (1/4 Angus, 1/4 Hereford, 1/4 Pinzgauer, and 1/4 Red Poll) and 75% Red Angus] from the beef physiology herd located at the Eastern Nebraska Research, Extension, and Education Center (ENREEC) were used in this study. Estrous cycles in cows were synchronized using two injections of prostaglandin F2alpha (PGF2a; 25 mg/mL; i.m.; Lutalyse, Zoetis Animal Health, Parsippany, NJ) 14 days apart. Heat patches were placed on tailheads at the last injection of PGF2a, and cows were determined to be in standing estrous by heat detection, and those that had 80% of heat detection patches activated. Estrus was considered day 0, and PGF2a injections were administered on days 9–11, at the time a mid-cycle corpus luteum was present, as determined via ultrasonography. Ovaries were collected via high lumbar ovariectomy as previously reported and described^31^ at 12 and 24 h after a third PGF2a injection on days 9–11 and the follicular granulosa (*n* = 6) and theca cells (*n* = 6) were isolated from the largest and second largest follicles. In brief, follicular granulosa cells from dominant antral follicles were suspended in DMEM/F12 culture media. After the granulosa cells were removed, the theca interna was removed with fine forceps. Granulosa cells were washed by centrifugation three times at 150 × *g* for 5–10 min and filtration through a 70 μm nylon mesh. The theca interna were suspended in collagenase 2 (103 IU/mL, Atlanta Biologicals) in DMEM/ F12 and dispersed using constant agitation at 37°C for 1 h. Dispersed theca cells were removed from the undigested tissue by filtration through a 70 μm mesh, then washed by centrifugation three times at 150 × *g* for 5–10 min.

### 2.3 | Luteal cell isolation

For luteal cell isolation, bovine ovaries were collected at a local slaughterhouse, and mid-cycle non-pregnant corpora lutea were staged as described.^32^ Uteri were checked for the presence of a fetus or visible gross abnormalities. The ovaries were immersed in 70% ethanol and then transported to the laboratory at 4°C in PBS. Using sterile technique, the corpus luteum was surgically dissected from the ovary and finely minced and dissociated using collagenase (103 U/ mL) in basal medium [M199 supplemented with antibiotics (100 U/mL penicillin G-sodium, 100 μg/mL streptomycin sulfate, and 10 μg/mL gentamicin sulfate)] for 45 min in spinner flasks at 35°C. The supernatant was transferred to a sterile 15 mL culture tube, washed three times with sterile PBS, and re-suspended in 10 mL of elutriation medium (calcium-free DMEM medium, 4.0 g/L glucose, antibiotics, 25 mM HEPES, 0.1% BSA, and 0.02 mg/mL deoxyribonuclease l; pH 7.2) on ice. Fresh dissociation medium was added to the remaining undigested tissue, incubated with agitation for an additional 45 min and processed as described above. The viability of cells was determined using trypan blue and cell concentration was estimated using a hemocytometer prior to cell elutriation.

Freshly dissociated cells were re-suspended in 15 mL of elutriation medium (calcium-free DMEM, 25 mM HEPES, 0.1% BSA, 0.02 mg/mL deoxyribonuclease, 3.89 g/L sodium bicarbonate, 3 mg/mL glucose, and antibiotics). Dispersed luteal cells were enriched for small and large luteal cells using a Beckman Coulter Avanti J-20 XP centrifuge equipped with a Beckman JE-5.0 elutriator rotor. The eluates were collected through continuous flow as previously described.^33^ In brief, a 100-mL fraction (F1) composed of erythrocytes and endothelial cells was obtained using a flow rate of 16 mL/min at 1800 RPM. Subsequently, the next 100 mL fraction (F2) was collected at a flow rate of 16 mL/min at 1400 RPM, which contained small luteal cells. A third fraction (F3) was collected using a flow rate of 24 mL/min at 1200 RPM. The remaining fraction (F4) was obtained at a flow rate of 30 mL/min at 680 RMP and was characterized by a high enrichment of large luteal cells. Cells were pelleted and resuspended in M199. Cells from fractions F2 and F4 were utilized in the experiments described. The viability, concentration, size of cell, and purity (%) in each fraction were determined using a hemocytomer and the trypan blue exclusion test. Cells with a diameter of 15–25 μm were classified as small luteal cells (purity of >90% enriched small luteal cells), and cells with a diameter >30 μm were classified as large luteal cells (purity of 70%–90% enriched large luteal cells).^34,35^

### 2.4 | Differentiation of ovarian follicular cells

Bovine granulosa (1 × 10^6^ cells/well) and theca cells (1 × 10^6^ cells/well) were cultured in a 6-well dish overnight in DMEM/F12 medium containing 1% fetal calf serum and antibiotics (100 U/mL of penicillin and 100 μg/mL of streptomycin). Cells were then cultured for up to four days in fresh DMEM/F12 medium containing 1% fetal calf serum and antibiotics or differentiation medium [DMEM/ F12, 1% fetal calf serum, insulin (10 mg/L), transferrin (5.5 mg/L), sodium selenium (6.7 μg/L), forskolin (FSK; 10 μM; adenylyl cyclase activator), phorbol myristate acetate (PMA, 20 nM; PKC/MAPK activator), and antibiotics]. On day two of treatment, cells were washed twice and incubated for 48 h in fresh differentiation media.

### 2.5 | Cell culture preparation and treatment with luteinizing hormone (LH)

Luteal cell cultures were plated in 12-well culture dishes at 5 × 10^5^ cells/well or 24-well culture dishes at 2.5 × 10^5^ cells/well. Cells were cultured overnight in culture media [M199 supplemented with 5% FBS, 0.1% BSA, and antibiotics] at 37°C in an atmosphere of 95% humidified air and 5% CO_2_.

Before treatments, cells were rinsed with PBS, and fresh serum-free culture medium was placed on cells and equilibrated at 37°C in an atmosphere of 95% humidified air and 5% CO_2_ for 2 h. Cells were treated with culture medium alone or LH (10 ng/mL) for 4 h at 37°C in an atmosphere of 95% humidified air and 5% CO_2_.

### 2.6 | Microarray

We mined bovine gene expression arrays from the NCBI GEO repository (GSE83524) to analyze the expression of the lipid droplet coat proteins PLINs 1–5s in freshly isolated bovine granulosa (GC, *n* = 4) and theca (TC, *n* = 3) cells from large follicles and from purified preparations of small (SLC, *n* = 3) and large (LLC, *n* = 3) bovine luteal cells from mature corpora lutea. Details of the isolation and analysis were previously published.^36,37^ Significant differences were identified as changes greater than 1.5-fold between GC and LLC or between TC and SLC, which were supported by unpaired *t*-tests with *p* < .01. Levels of *ACTB* mRNA were not significantly different among cell types. When all cell types were combined as a group, the relative expression of *ACTB* mRNA was 7845 ± 164, mean ± *SEM*.

### 2.7 | siRNA knockdown of PLIN2

PLIN2 was knocked down using silencing RNA (siRNA) to determine the effects of PLIN2 on progesterone production. In brief, enriched small luteal cell populations were transfected with siControl or PLIN2 siRNA (75 nM) for 6 h using Lipofectamine RNAimax in an opti-M EM1 culture medium. Following transfection, 5% FBS was added to the culture medium, and incubations were continued for 48 h. Successful knockdown of PLIN2 was confirmed by Western blot for each experiment. Following knockdown of PLIN2, the medium was changed, and cells were equilibrated for 2 h before treatment with LH (10 ng/mL) for 4 h. Conditioned medium and cell lysates were immediately collected and stored at −20°C until further analysis.

### 2.8 | Treatment with adenoviruses

The adenoviruses expressing β-galactosidase (Ad.βGal; prepared by Chris Wolford, Ohio State University, Columbus, Ohio) were previously described.^38–40^ In brief, enriched small luteal cells were seeded into 12-well culture dishes and maintained at 37°C in an atmosphere of 95% humidified air and 5% CO_2_ for 24 h before adenoviral infection. Ad.βGal, Ad.PLIN2, or Ad.PLIN3 were added to cell cultures in a serum-free culture medium. After 2 h, the media was replaced with M199 enriched with 5% FBS and maintained for an additional 48 h at 37°C in an atmosphere of 95% humidified air and 5% CO_2_. The medium was changed, and cells were equilibrated for 2 h before treatment with the control medium or LH (10 ng/ mL; 4 h). Following treatment, the protein was extracted, quantified, and subjected to Western blotting, or cell cultures were prepared for confocal microscopy.

### 2.9 | Western blotting analysis

Following incubation, cells were immediately placed on ice and rinsed three times with 1 mL of ice-cold PBS. Cells were lysed with 100 μL cell lysis buffer and removed from the culture dish using a cell scraper for sonication at 40% power setting (VibraCell, Model CV188) as previously described.^41^ Protein concentrations were determined using a Bradford protein assay (Bio-Rad Protein Assay). Samples were adjusted with water to ensure equal protein concentrations and then suspended in 6× Laemmli buffer before being placed on a dry heat bath at 100°C for 6 min.

Proteins (20 μg/sample) were resolved using 10% SDS-PAGE or 4%–20% Mini-PROTEAN^®^ TGX^™^ precast protein gel and then transferred to nitrocellulose membranes. Membranes were blocked with Tris-buffered saline + 0.1% Tween-20 (TBS-T) containing a 5% non-fat milk solution at room temperature for 1 h. Membranes were incubated with primary antibodies (Table 1) for 24 h at 4°C for the detection of total and phosphorylated proteins. Membranes were rinsed three times with TBS-T for 5 min. Membranes were then incubated with an appropriate horseradish peroxidase-linked secondary antibody (Table 1) for 1 h at room temperature. Blots were then rinsed with TBS-T, and chemiluminescent substrate was applied per the manufacturer’s instructions. Blots were visualized using a UVP Biospectrum 500 Multi-Spectral Imaging System (UVP, Upland, CA, USA), and the percent abundance of immunoreactive protein was determined using densitometry analysis in VisionWorks (UVP). Total proteins were normalized to a beta-actin or beta-tubulin prior to the calculation of fold induction. Fold increases due to treatment were then calculated.

### 2.10 | Immunohistochemistry

Bovine ovaries were sliced, and portions were fixed in 10% formalin for 24 h and then changed into 70% ethanol until embedded in paraffin. Tissues were cut into 4 μm sections and mounted onto polylysine-coated slides. Slides were deparaffinized through three changes of xylene and through graded alcohols to water and microwaved in an unmasking solution (Vector H-3300) for antigen retrieval. Endogenous peroxidase was quenched with 0.3% hydrogen peroxide in methanol for 30 min. Sections were incubated with anti-PLIN2 overnight at 4°C, as indicated in Table 1, and subsequently with anti-guinea pig HRP for 1 h at room temperature. Slides were counterstained with Mayer’s hematoxylin, dehydrated through graded alcohols, and mounted with Fluoromount-G. Non-immune IgG from the host species was used as a control.

### 2.11 | Progesterone analysis

Progesterone concentrations from conditioned culture media were determined using a commercially available ELISA kit per the manufacturer’s protocol (intra-assay CV = 4.83%; inter-assay = 12.02%). The analytical sensitivity of the kit is 0.045 ng/mL.

### 2.12 | Confocal microscopy

For all confocal microscopy experiments, sterile No. 1.5 glass coverslips (22 × 22 mm) were individually placed in each well of a 6-well culture dish. Enriched small luteal cell cultures were seeded at 5 × 10^5^ cells/well.

To determine the effects of exogenous, PLIN2 or PLIN3 proteins on lipid droplet number and volume, the adenoviruses Ad.βGal (control virus), Ad.PLIN2, or Ad.PLIN3 were added to cell cultures in a serum-free culture medium. After 2 h, the media was replaced with M199 enriched with 5% FBS and maintained for an additional 48 h at 37°C in an atmosphere of 95% humidified air and 5% CO_2_.

Cells were fixed with 200 μL of 4% paraformaldehyde and incubated at 4°C for 30 min. Cells were rinsed 3× with 1 mL 1× PBS following fixation and then incubated with 200 μL of 0.1% Triton-X in 1× PBS-T (0.1% Tween-20) at room temperature for 10 min to permeabilize the membranes. The permeabilized cells were rinsed 3× with PBS and then blocked in 5% BSA for 24 h at 4°C. Cells were then rinsed, and appropriate antibodies (Table 1) were added to each coverslip and incubated at room temperature for 60 min. Following incubation, cells were rinsed 3× with PBS to remove the unbound antibody. Cells were then incubated with appropriate secondary antibodies (Table 1) at room temperature for 60 min. Cells were rinsed 3× with 1 mL 1× PBS to remove unbound antibodies. Following labeling with antibodies, coverslips containing labeled cells were mounted to glass microscope slides using 10 μL Fluoromount-G (Electron Microscopy Sciences). Coverslips were sealed to glass microscope slides using clear nail polish and stored at −22°C until imaging.

To determine the effects of exogenous PLIN2 or PLIN3 proteins on lipid droplet number and volume, images were collected using a Zeiss 800 confocal microscope equipped with a 63× oil immersion objective (1.4 N.A) and an acquisition image size of 1024 × 1024 pixels (101.31 μm × 101.31 μm). The appropriate filters were used to excite each fluorophore, and the emission of light was collected between 450 and 1000 nm. Cells were randomly selected from each slide, and *z*-stacked (0.33 μm) images were generated from bottom to top of each cell. *Z*-stacked images were converted to maximum intensity projections and processed utilizing ImageJ (RRID:SCR_003070; National Institutes of Health) analysis software. To determine the size and number of lipid droplets in cells infected with Ad.PLINs, images were quantified with ImageJ using the AnalyzeParticles function in threshold images, with size (square pixel) settings from 0.1 to 100 and circularity from 0 to 1. Outputs were then converted into microns.

### 2.13 | Part II: In vivo analysis following Prostaglandin F2α

#### 2.13.1 | Cattle

Post-pubertal, non-lactating multiparous female cattle of composite breeding [25% MARC III (1/4 Angus, 1/4 Hereford, 1/4 Pinzgauer, 1/4 Red Poll) and 75% Red Angus] beef cows from the beef physiology herd at the Eastern Nebraska Research and Extension Center (ENREC) were used in this study. Cows were synchronized using two intramuscular injections of PGF2α (25 mg; Lutalyse^®^) 11 days apart, as described in Part I. At mid-cycle (days 9–10), cows were treated with an intra-muscular injection of saline or PGF2α (25 mg). At each of the four time-points post injection (0, 4, 12, and 24 h), cows were subjected to a bilateral ovariectomy through a right flank approach under local anesthesia as previously described.^31,42,43^ The corpus luteum was removed from each ovary, weighed, and <5 mm^3^ sections were snap frozen in liquid N_2_ for subsequent protein analysis or fixed in 10% formalin for immunohistochemistry. The University of Nebraska-Lincoln Institutional Animal Care and Use Committee approved all procedures and facilities used in this animal experiment, and animal procedures were performed at the University of Nebraska—Lincoln, Animal Science Department.

### 2.14 | Progesterone analysis

Plasma progesterone concentrations were determined using a radioimmunoassay (RIA) to detect progesterone concentrations as previously described.^44^ Progesterone concentrations were determined using the ImmuChemTM Coated Tube Progesterone 125I RIA kit (intra-assay CV = 2.0%, inter-assay CV = 4.46%). The sensitivity of the kit is 0.02 ng/mL.

### 2.15 | RNA sequencing

We mined bovine RNA sequencing data from the NCBI GEO repository (GSE217053) to analyze the expression of the lipid droplet coat proteins PLINs 1–5 s in corpora lutea obtained from mid-luteal phase cows (Day 10; *n* = 6) and corpora lutea obtained from animals treated with 4 (*n* = 6) and 12 h (*n* = 6) i.m administration of PGF2α. Details of the RNA isolation and analysis were previously published.^45^

### 2.16 | Western blotting

Approximately 100 mg of tissue was homogenized in RIPA Buffer supplemented with 1× Halt Protease and Phosphatase Inhibitor Cocktail and sonicated at 40% power setting (VibraCell, Model CV188) as previously described.^41^ Following sonification, tissue homogenates were centrifuged at 13000 × *g* at 4°C for 15 min. Protein was collected, and concentrations were determined using the BCA protein assay. Samples were suspended in 6× Laemmli buffer and placed on a dry heat bath at 100°C for 6 min. Proteins (30 μg/sample) were resolved and visualized as described in Part 1. Total PLIN2 was normalized to ACTB.

### 2.17 | Statistics

Each experiment was performed at least three times, each using cell preparations from separate cows and dates of collection. Specifics of statistical testing are described in the relevant figure legends. The differences in means were analyzed by one-way ANOVA followed by Tukey’s multiple comparison tests to evaluate multiple responses, one-way ANOVA followed by Dunnett’s posttests to compare means, or by *t*-tests to evaluate paired responses. A two-way ANOVA was used to evaluate repeated measures with Dunnett’s posttests to compare means. All statistical analysis was performed using GraphPad Prism software (GraphPad Prism, RRID:SCR_002798). All data are presented as the means ± *SEM*.

## 3 | RESULTS

### 3.1 | Perilipin 2 (PLIN2) expression in the bovine ovary

Following ovulation, during the follicular to luteal transition, the granulosa and theca cells of the ovarian follicle differentiate to form the large and small luteal cells, respectively, of the bovine corpus luteum.^46^ We mined bovine gene expression arrays from the NCBI GEO repository (GSE83524) to analyze the expression of the lipid droplet coat proteins PLINs 1–5 s in freshly isolated bovine granulosa (GC, *n* = 4) and theca (TC, *n* = 3) cells from large follicles and from purified preparations of small (SLC, *n* = 3) and large (LLC, *n* = 3) bovine luteal cells from mature corpora lutea. Figure S1 shows the relative mRNA expression of *PLIN1*, *PLIN2*, *PLIN3*, *PLIN4*, *and PLIN5* in follicular cells and their luteal cell counterparts. Levels of mRNA expression for *PLIN1*, *PLIN4*, and *PLIN5* were low and not different among cell types. Transcripts for *PLIN2* and *PLIN3* were similar in granulosa and theca cells. Levels of *PLIN2* were increased 3.0-fold in large luteal cells compared to granulosa cells (*p* < .001) and increased 4.4-fold in small luteal cells when compared to theca cells (*p* < .001). Levels of *PLIN3* were not influenced in large luteal cells compared to granulosa cells (*p* > .05) but were 2-fold greater in small luteal cells when compared to theca cells (*p* < .001). Levels of ACTB mRNA were not significantly different among cell types. When all cell types were combined, the relative expression of the ACTB mRNA count was 7845 ± 164, mean ± *SEM*.

**FIGURE 1:**
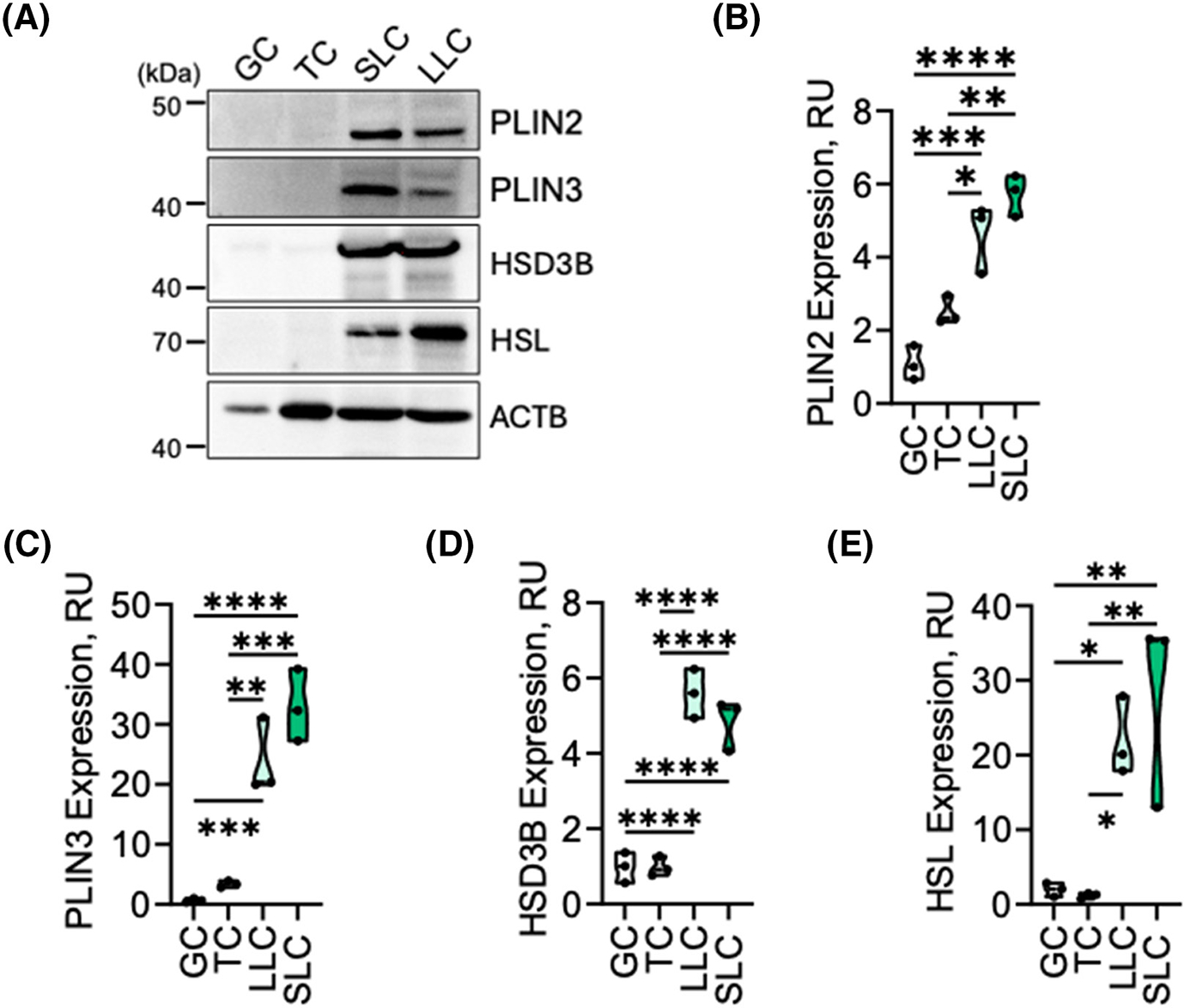
PLIN2 is highly enriched in bovine luteal cells. Western blotting was used to determine validated changes in expression of PLIN2 and PLIN3 in freshly isolated bovine granulosa (GC) and theca cells (TC) from large follicles and purified preparations of bovine small and large luteal cells from mature corpora lutea. (A) Representative Western blot of PLIN2, PLIN3, HSD3B, and HSL expression (*n* = 3). (B) Quantitative analysis of PLIN2 expression. (C) Quantitative analysis of PLIN3 expression. (D) Quantitative analysis of HSD3B expression. (E) Quantitative analysis of HSL expression. Statistics were performed by one-way ANOVA, followed by Tukey’s multiple comparison tests. Data are means ± standard error, *n* = 3. Bars represent means ± *SEM*, *n* = 3. Significant difference between treatment, *p < .05; **p < .01; ***p < .001; ****p < .0001. Perilipin 2 (PLIN2); Perilipin 3 (PLIN3); 3beta-Hydroxysteroid dehydrogenase (HSD3B); Hormone Sensitive Lipase (HSL); Beta-Actin (ACTB; loading control).

Western blot was used to validate expression of PLIN2, PLIN3, and 3beta-Hydroxysteroid dehydrogenase (HSD3B) in granulosa, theca, and their respective luteal cell counterparts (Figure 1A). Protein expression for PLIN2, PLIN3, HSD3B, and HSL was similar in granulosa and theca cells (*p* > .05). Levels of PLIN2 were increased 4.2-fold in large luteal cells compared to granulosa cells (*p* < .001) and increased 2.2-fold in small luteal cells when compared to theca cells (*p* < .001; Figure 1A,B). Levels of PLIN3 were increased 34.8-fold in large luteal cells compared to granulosa cells (*p* < .001) and 9.7-fold in small luteal cells when compared to theca cells (*p* < .001; Figure 1A,C). Levels of HSD3B were increased 5.7-fold in large luteal cells compared to granulosa cells (*p* < .0001) and increased 4.9-f old in small luteal cells when compared to theca cells (*p* < .0001; Figure 1A,D). Levels of HSL were increased 11.1-fold in large luteal cells compared to granulosa cells (*p* < .05) and 23.9-fold in small luteal cells when compared to theca cells (*p* < .01; Figure 1A,E).

### 3.2 | Perilipin 2 (PLIN2) expression following follicular cell differentiation

**FIGURE 2:**
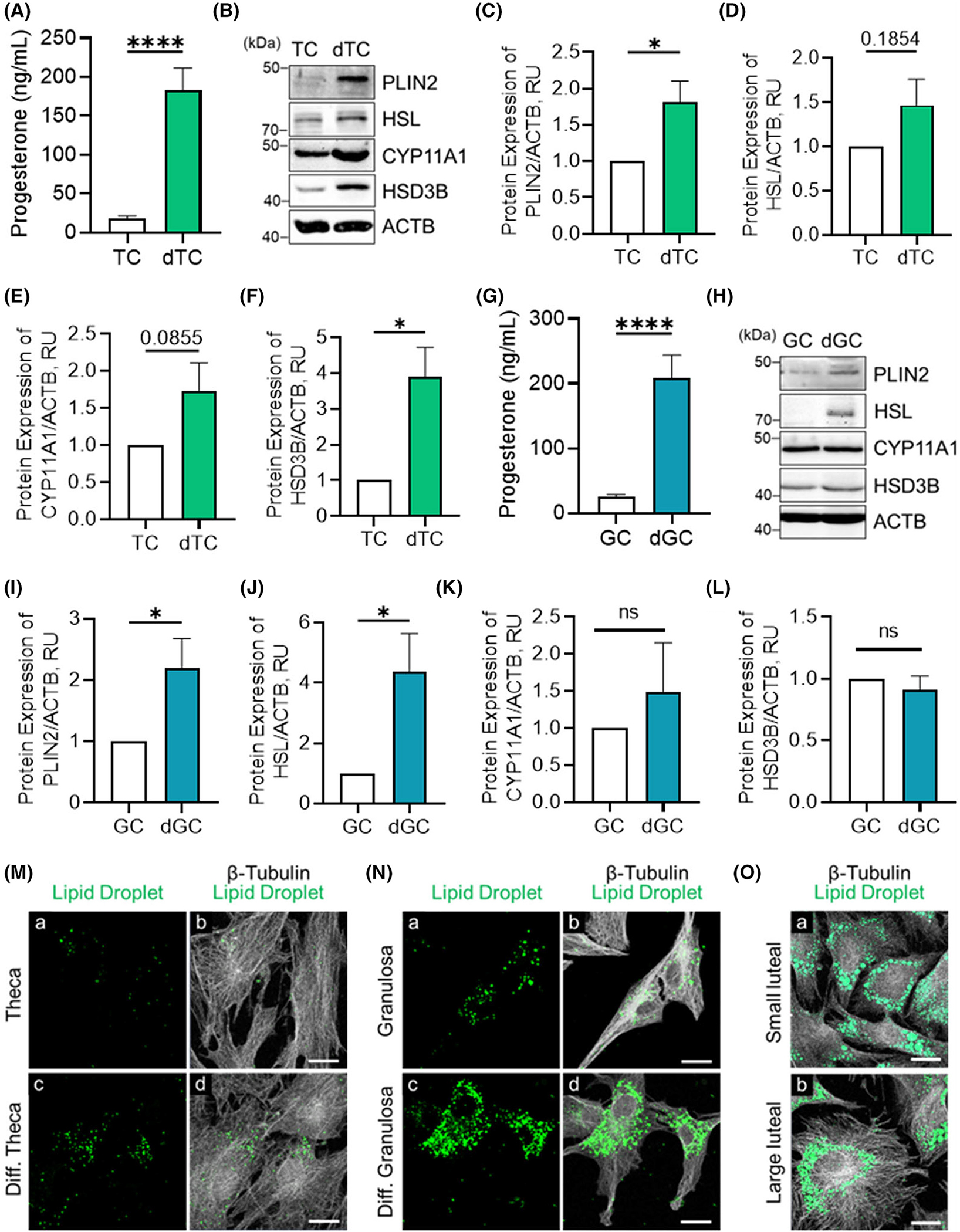
PLIN2 expression following follicular cell differentiation. Bovine granulosa (GC) and theca cells (TC) were cultured for up to four days in medium containing 1% fetal calf serum with or without insulin/transferrin/selenium, the adenylyl cyclase activator forskolin (10 μM), and phorbol myristate acetate (PMA, 20 nM). (A) Medium progesterone obtained from TC following incubation with or without differentiation media. (B) Representative Western blot of PLIN2, HSL, CYP11A1, and HSD3B expression in TC and differentiated TCs (dTC; *n* = 4–6). (C) Quantitative analysis of PLIN2 expression. (D) Quantitative analysis of HSL expression. (E) Quantitative analysis of CYP11A1 expression. (F) Quantitative analysis of HSD3B expression. (G) Medium progesterone obtained from GCs following incubation with or without differentiation media. (H) Representative Western blot of PLIN2, HSL, CYP11A1, and HSD3B expression in GC and differentiated TCs (dGC; *n* = 6). (I) Quantitative analysis of PLIN2 expression. (J) Quantitative analysis of HSL expression. (K) Quantitative analysis of CYP11A1 expression. (L) Quantitative analysis of HSD3B expression. (M) Representative micrographs of lipid droplets obtained from TC (panels a and b) and dTC (panels c and d). (N) Representative micrographs of lipid droplets obtained from GC (panels a and b) and dGC (panels c and d). (O) Representative micrographs of lipid droplets obtained from small luteal (panel a) and large luteal cells (panel b). The micron bar represents 20 μm. Statistics were performed using *t*-tests to evaluate paired responses. Bars represent means ± *SEM*. Significant difference between treatments, *p < .05; **p < .01. Perilipin 2 (PLIN2); hormone sensitive lipase (HSL); cholesterol side-chain cleavage enzyme (CYP11A1); 3beta-hydroxysteroid dehydrogenase (HSD3B); and beta actin (ACTB; loading control).

The differentiation of ovarian cells into their respective luteal cells is driven by mimicking the surge of LH, which activates PKA and PKC signaling.^47^ To determine the effects of cellular differentiation on PLIN2 expression, theca and granulosa cells were stimulated with FSK (10 μM), an activator of PKA signaling, PMA (20 nM), an activator of PKC signaling, and 1× ITS for 96 h, and protein lysis and conditioned media were collected. Differentiation of theca cells for 96 h stimulated a 9.8-fold increase in progesterone production (*p* < .05; Figure 2A). Western blotting was used to determine changes in protein expression following theca cell differentiation (Figure 2B). Western blotting revealed that differentiation of theca cells increased the expression of PLIN2 1.8-f old compared to untreated theca cells (*p* < .05; Figure 2B,C). Although we observed an increase in the expression of HSL in small luteal cells compared to theca cells (Figure 1A,E), we did not observe a significant difference in HSL expression following incubation with differentiation media (*p* > .05; Figure 2B,D). Western blotting further revealed a tendency for cholesterol side-chain cleavage enzyme (CYP11A1) expression (*p* = .0855; Figure 2B,E) and an increase in HSD3B expression (*p* < .05; Figure 2B,F) in differentiated theca cells when compared to untreated theca cells.

Next, we examined the effects of differentiation in granulosa cells. Differentiation of granulosa cells for 96 h stimulated an 8.4-fold increase in progesterone production (*p* < .01; Figure 2G). Western blotting revealed differentiation of granulosa cells increased the expression of PLIN2 2.1-fold compared to untreated granulosa cells (*p* < .05; Figure 2H,I). Moreover, we observe a 4.3-f old increase in HSL expression following incubation with differentiation media (*p* < .05; Figure 2H,J), but no difference in the expression of steroidogenic enzymes CYP11A1 (*p* > .05; Figure 2H,K) and HSD3B (*p* > .05; Figure 2H,L).

Confocal microscopy was used to visualize the lipid droplet content between the differentiated and untreated theca (Figure 2M) and granulosa cells (Figure 2N). Untreated theca (Figure 2M panels a and b) and granulosa cells (Figure 2N panels a and b) had fewer lipid droplets than the differentiated follicular cells (*p* > .05; Figure 2M,N panels c and d; Figure S2), as assessed by BODIPY staining of lipid droplets. Lipid droplet content was also visualized in small and large luteal cells as a comparison (Figure 2O).

### 3.3 | PLIN2 knockdown increases acute progesterone production in small luteal cells

**FIGURE 3:**
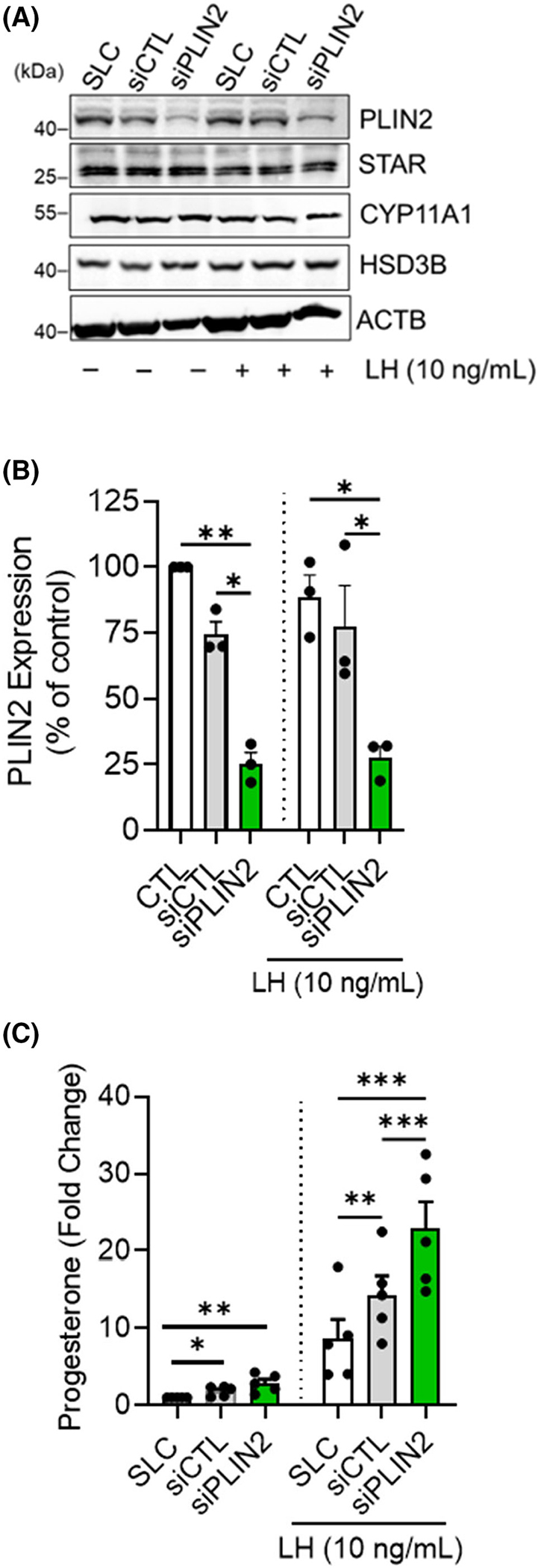
Knockdown of lipid droplet-associated proteins, PLIN2, promotes acute progesterone production in bovine small luteal cells. PLIN2 mRNA was silenced using siPLIN2 in small bovine luteal cells. Following knockdown, cells were treated without (control; CTL) or with luteinizing hormone (LH; 10 ng/ mL) for 4 h. (A) Representative Western blot analysis showing expression of PLIN2 in siPLIN2 knockdown small luteal cells. (B) Quantitative analysis of the expression of PLIN2 in siPLIN2 knockdown small luteal cells. (C) medium progesterone. Statistics were performed by a two-way ANOVA, which was used to evaluate repeated measures with Tukey’s multiple comparison tests. Bars represent means ± *SEM*, *n* = 3. Significant difference between treatments, *p < .05; **p < .01; ***p < .001. Steroidogenic acute regulatory protein (STAR); cholesterol side-chain cleavage enzyme (CYP11A1); 3beta-hydroxysteroid dehydrogenase (HSD3B); beta-actin (ACTB; loading control).

We employed specific siRNA targeting of PLIN2 to evaluate the role of PLIN2 on progesterone production in small luteal cells (Figure 3A). Western blotting revealed a 75 ± 5.6% decrease in expression of PLIN2 in siPLIN2-treated luteal cells compared to control cells (*p* < .05; Figure 3B). Treatment of small luteal cells with siPLIN2 did not significantly influence the expression of steroidogenic enzymes (*p* > .05; data not shown). Treatment of small luteal cells with siPLIN2 did not significantly influence basal progesterone secretion when compared to siControl cells (*p* > .05; Figure 3C). However, siRNA-mediated knockdown of PLIN2 increased LH-induced progesterone secretion compared to siControl treated cells (*p* < .05; Figure 3C).

### 3.4 | Overexpression of Perilipin 2 (PLIN2) and Perilipin 3 (PLIN3) attenuates progesterone production in small luteal cells

**FIGURE 4:**
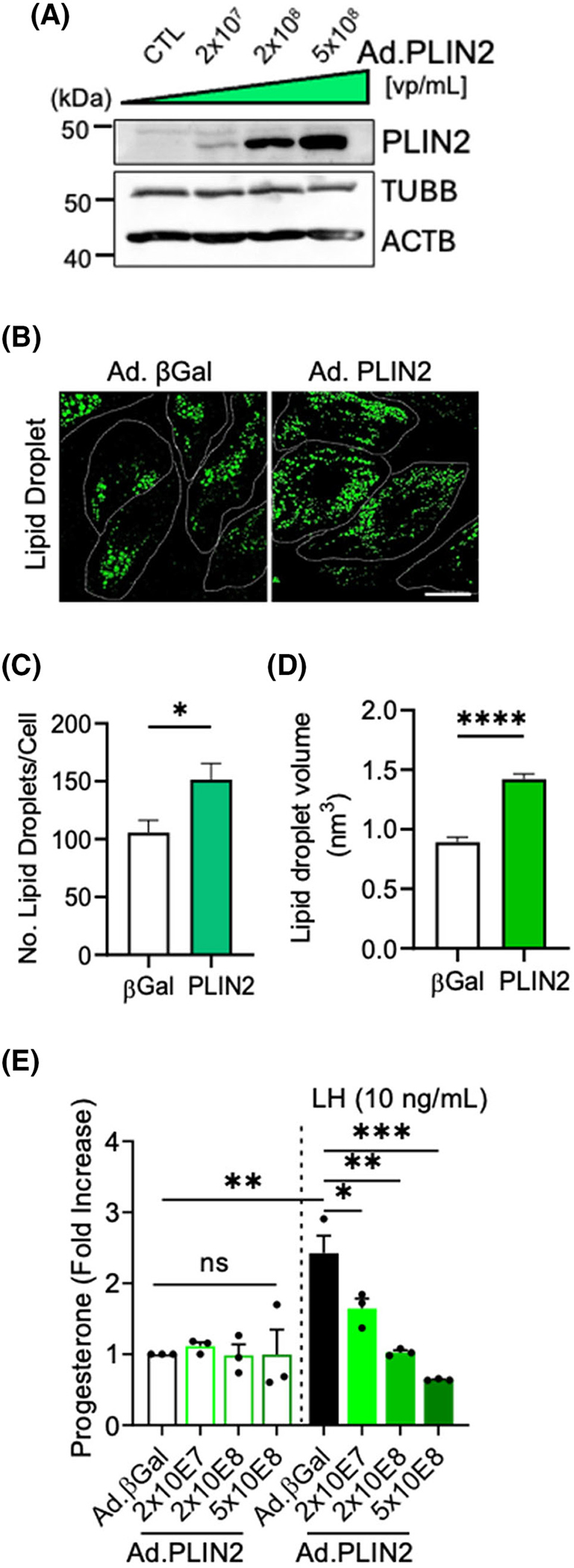
Overexpression of lipid droplet-associated proteins, PLIN2, in bovine luteal cells. Replication-deficient adenoviruses (Ad) containing beta-galactose (Ad.βGal; control) or PLIN2 (Ad. PLIN2) were utilized to overexpress PLIN2 in bovine small luteal cells. (A) Representative Western blot of dose-dependent overexpression of Ad.PLIN2 [VP/mL] in small luteal cells. Small luteal cells were infected with Ad.βGal or Ad.PLIN2 as described above. After 48 h, luteal cells were equilibrated for 2 h and stimulated with luteinizing hormone (LH; 10 ng/mL) for 4 h. Small luteal cells were treated with Ad.βGal or Ad.PLIN2 and lipid droplets were labeled (Lipi-blue 1 μM) and visualized by confocal microscopy. (B) Representative micrographs of lipid droplets obtained from small luteal cells infected with Ad.βGal or Ad.PLIN2 (2х10^8^ VP/mL). (C) Quantification of lipid droplet number in small luteal cells infected with Ad.βGal or Ad.PLIN2. (D) Quantification of lipid droplet volume (nm^3^) in small luteal cells infected with Ad.βGal or Ad.PLIN2. Statistics were performed by *t*-tests to evaluate paired responses. Data are means ± standard error, *n* = 3. (E) Medium progesterone obtained from small luteal cells treated with Ad.βGal or increasing concentrations Ad.PLIN2 following stimulation with LH. Statistics were performed by two-way ANOVA was used to evaluate repeated measures with Tukey’s multiple comparison tests. Bars represent means ± *SEM*, *n* = 3. Significant difference between treatments, *p < .05; **p < .01; ***p < .001; ****p < .0001. Micron bar represents 20 μm. Beta Actin (ACTB; loading control); Beta Tubulin (TUBB; loading control).

The bovine steroidogenic luteal cells express mRNA for both PLIN2 and PLIN3 (Figure S1). Therefore, we set out to determine the role of overexpression of PLIN2 and PLIN3 on lipid droplet abundance and progesterone production in small luteal cells. To determine the role of overexpression of PLIN2 on acute progesterone production, luteal cells were infected with increasing concentrations of adenovirus (Ad) expressing PLIN2, and Ad.β-Gal is used as a control. (Figure 4). Western blotting revealed that Ad.PLIN2 increased the expression of PLIN2 in small luteal cells (Figure 4A). We set out to determine the effects of exogenous PLIN2 expression on lipid droplet number and volume in small luteal cells (Figure 4B). Overexpression of PLIN2 induced a 1.6-fold increase in the number of lipid droplets (*p* < .05; Figure 4C) and a 1.4-fold increase in average lipid droplet volume (*p* < .0001; Figure 4D) when compared to cells treated with Ad.β-Gal control. To examine the effects of exogenous PLIN2 expression on progesterone production, enriched populations of small luteal cells were treated with increasing concentrations of Ad.PLIN2 and then stimulated with or without LH (10 ng/mL) for 4 h. We observed no difference in basal progesterone production in cells treated with Ad.PLIN2 compared to Ad.β-Gal control (Figure 4E). There was a concentration-dependent decrease in acute progesterone production in cells treated with increasing titers of Ad.PLIN2, when compared to the Ad.β-Gal control cells following stimulation with LH (*p* < .05; Figure 4E).

Increasing concentrations of adenovirus expressing PLIN3 lead to a concentration-dependent increase in the expression of PLIN3 in small luteal cells (Figure S3A). We determined the effects of exogenous PLIN3 expression on lipid droplet number and volume in small luteal cells (Figure S3B). Overexpression of PLIN3 increased lipid droplet volume (*p* < .05; Figure S3C) and induced a tendency to reduce the number of lipid droplets (*p* = .09; Figure S3D) when compared to cells infected with Ad.β-Gal control. We observed no difference in basal progesterone production in cells treated with Ad.PLIN3 compared to Ad.β-Gal control (*p* > .05; Figure 3E). However, a decrease in LH-stimulated progesterone production was observed in cells infected with Ad.PLIN3 when compared to Ad.β-Gal control cells (*p* < .05; Figure S3E).

### 3.5 | Effects of Prostaglandin F2 alpha on Perilipin 2 (PLIN2) expression in vivo

**FIGURE 5:**
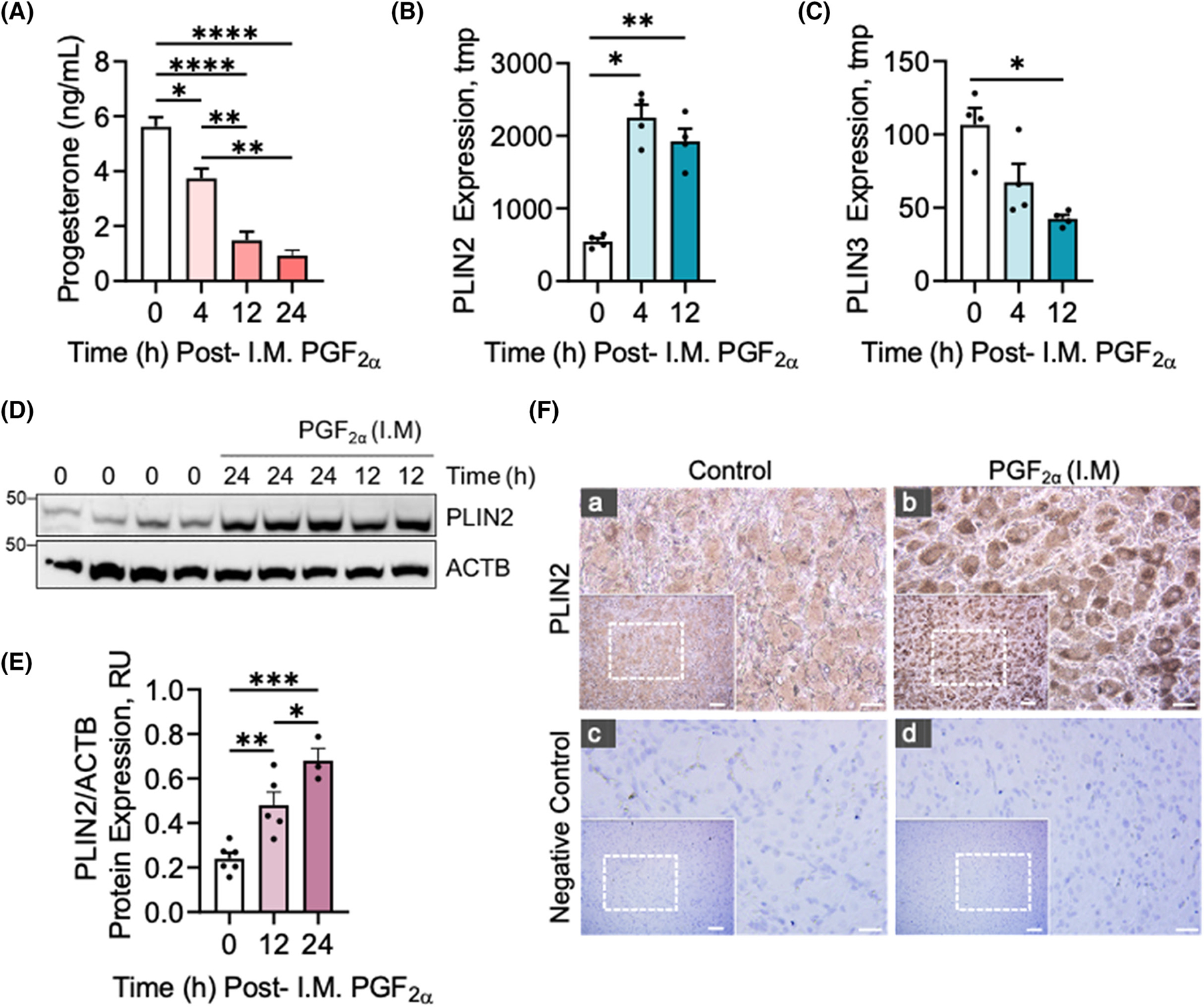
Effects of Prostaglandin (PG) F2α on PLIN2 expression in vivo. Midcycle cows (*n* = 3–8/time-point) were administered I.M. Prostaglandin F2α (PGF2α; 25 mg) for 4, 12, and 24 h or control saline injections. (A) Serum progesterone concentrations were obtained from animals 0 (*n* = 8), 4 (*n* = 3), 12 (*n* = 6), and 24 h (*n* = 3) following I.M PGF2α administration. Statistics were performed by one-way ANOVA followed by Tukey’s multiple comparison tests. Mid-luteal phase cows were injected with saline (Control) or PGF2α, (25 mg, i.m.) and ovariectomized after 4 and 12 h to collect corpora lutea. RNA sequencing of whole luteal tissue was performed. (B) mRNA levels of PLIN2 in the bovine corpus luteum at midcycle and 4- and 12-h post-PGF2α injection. (C) mRNA levels of PLIN3 in the bovine corpus luteum at midcycle and 4- and 12-h post-PGF2α injection. Data are presented as mean number of transcripts per million (TPM) ± *SEM*. *n* = 4; *p < .05, **p < .01, compared to 0 h by DESeq2 analysis, Benjamini Hochberg correction. P-values shown are adjusted p-values for multiple comparisons. (D) Representative Western blot of PLIN2 expression in bovine corpus luteum at midcycle and 12- and 24 h post-PGF2α injection. (E) Quantitative analysis of PLIN2 expression following 0 (*n* = 6), 12 (*n* = 5) and 24 h (*n* = 3) post-PGF2α injection. Statistics were performed by one-w ay ANOVA followed by Tukey’s multiple comparison tests. (F) Representative immunohistochemistry micrograph of the PLIN2 in luteal tissue 12 h following I.M administration of PGF2α treatment. Micron bar = 5 mm (Insert) and 1 mm (Enlarged). Bars represent means ± *SEM*. Significant difference between treatments, *p < .05; **p < .01; ***p < .001; ****p < .0001.

To evaluate the temporal effects of PGF2α on progesterone production, cows were administered a single dose of saline or PGF2α (i.m.) and corpora lutea were collected at zero time, 4, 12, and 24 h post i.m administration of PGF2α. Serum progesterone decreased 33.6% 4 h post-injection of PGF2α (*p* < .05) and further decreased 73.7 and 82.3%, respectively, 12 and 24 h post-injection of PGF2α (*p* < .0001; Figure 5A).

Next, we evaluated the effects of PGF2α on the gene expression of the PLIN^1–5^ family of lipid droplet coat proteins (Figures 5B,C and S4). RNA sequencing of mid-luteal phase (Day 10) and regressing corpora lutea (4 and 12 h) revealed a 4.1-fold increase at 4 h (*p* < .05) and a 3.5-fold increase in the number of *PLIN2* transcripts 12 h post i.m administration of PGF2α (*p* < .01; Figure 5B). In contrast, RNA sequencing revealed no difference in the mRNA transcripts of *PLIN1* (*p* > .05; Figure S4A), and a decrease in the mRNA transcripts of *PLIN3* (*p* < .05; Figure 5C), *PLIN4* (*p* < .01; Figure S4B), and *PLIN5* (*p* < .05; Figure S4C), 12 h post i.m administration of PGF2α (*p* < .05; Figure 5C).

Western blotting was used to confirm the observed increase in PLIN2 expression following i.m administration of PGF2α (Figure 5D). Western blot revealed an acute 1.9-fold increase (*p*-value < .01) in PLIN2 expression 12 h post-treatment with PGF2α and a 2.8-fold increase 24 h post-PGF2α, respectively (*p*-value < .001; Figure 5D,E). Immunohistochemistry of luteal tissue revealed an observed increase in PLIN2 (Figure 5F, panel a and b) 12 h post i.m administration of PGF2α. Moreover, there was a notable presence of PLIN2 localized to the steroidogenic large and small luteal cell populations (Figure 5F panels a and b), supporting our hypothesis that PGF2α regulates PLIN2 expression in bovine luteal tissue.

## 4 | DISCUSSION

Perilipins 1–5 are major coat proteins associated with lipid droplets that target and regulate intracellular lipid storage and hydrolysis.^48^ Specifically, the highly conserved 11-mer repeat regions of PLINs 1–3 form amphipathic helices on the lipid droplet surface and coordinate lipid release from these droplets.^49^ These organelles store cholesterol esters and are enriched with proteins that regulate both lipid homeostasis and steroid production in steroidogenic tissues, such as the corpus luteum.^2,5,50^ The small and large steroidogenic cells of the corpus luteum exhibit a unique abundance of cholesteryl ester-storing lipid droplets, which have been proposed to contribute to sex steroid synthesis.^5^ Our study focuses on PLIN2, a coat protein associated with lipid droplets, and examines its role in luteal cells and its impact on progesterone production. We report that PLIN2 is highly expressed in ovarian steroidogenic cells and correlates with follicular cell differentiation. Importantly, genetic manipulation of PLIN2 alters acute LH-stimulated progesterone production in small luteal cells. Additionally, our findings indicate that administering the luteolytic hormone, PGF2α, increases PLIN2 expression, a change that is accompanied by reduced serum progesterone levels.

The transformation of ovarian follicular granulosa and theca cells into steroidogenic luteal cells is a key aspect of corpus luteum development. By comparing follicular cells before and after differentiation, we gain insights into the cellular changes that occur during luteinization. The role of lipid droplets in energy storage and as precursors for steroid hormones is crucial, and the observed increase in PLIN2 expression, along with the accumulation of lipid droplets in differentiated cells, underscores their significance in the metabolic adaptation of luteal cells. This study enhances our understanding of the intricate processes of cellular differentiation, hormonal regulation, and metabolic shifts that are essential in luteal formation.

Steroidogenic cells synthesize steroids upon stimulation, requiring a steady cholesterol supply. Hormone stimulation, particularly LH via cAMP/PKA signaling, prompts lipid droplets to release cholesterol for steroid hormone synthesis, affecting processes like HSL phosphorylation and localization on lipid droplets.^10^ This mechanism aids in transporting cholesterol to mitochondria for progesterone biosynthesis in bovine luteal cells. Our findings indicate that PLIN2 knockdown enhances hormone-stimulated progesterone production, likely by enhancing HSL access and promoting cholesteryl ester hydrolysis, akin to the role of PLIN2 in adrenal tissue cholesterol flux.^51^ Conversely, overexpression of PLIN2 inhibits acute LH-stimulated progesterone production in small luteal cells, accompanied by an increase in the number and volume of lipid droplets per cell. A similar effect is seen in cardiac tissue^52^ and cultured fibroblasts,^53^ whereby overexpression of PLIN2 leads to an accumulation of intracellular lipid droplets and triacylglycerol content. Such overexpression of PLIN2 in adipocytes also impedes the association of adipose triglyceride lipase, a critical lipase responsible for the hydrolysis of triacylglycerol, with the lipid droplet.^54^ A similar mechanism involving HSL may be occurring in luteal cells, leading to a decrease in intracellular cholesterol available for steroidogenesis.

Lipid droplets and the lipid droplet-associated protein, PLIN2, are a predominant feature of the steroidogenic cells of the bovine corpus luteum.^5^ It is hypothesized that PLIN2 may function as a regulatory ‘brake’ regarding progesterone synthesis. Notably, under basal conditions, large luteal cells exhibit a greater capacity for progesterone synthesis compared to small luteal cells. Supporting this theory, microarray analysis of luteal cells shows higher expression of PLIN2 in small luteal cells compared to large luteal cells, hinting at a potential regulatory role. Knockdown of PLIN2 in small luteal cells enhances acute progesterone production, whereas overexpressing PLIN2 in small luteal cells suppresses synthesis, further indicating its inhibitory impact on steroidogenesis. This concept gains additional support following treatment with PGF2α. After such treatment, a significant increase in PLIN2 expression is observed, especially in large luteal cells, coinciding with a marked decrease in serum progesterone levels. These observations support the idea that PLIN2 may play a vital role in modulating progesterone synthesis, potentially serving as a key regulatory factor in hormonal control within the corpus luteum.

Bovine luteal cells express PLIN3, which translocates between the lipid droplet and cytoplasm.^55^ As the second most common perilipin in these cells,^5^ the exact role of PLIN3 in the corpus luteum is unclear. In neutrophils, PLIN3 is crucial for lipid droplet formation and prostaglandin E2 production,^56^ a luteotropic hormone also synthesized by the corpus luteum. Overexpressing PLIN3 in our study led to larger lipid droplets and reduced hormone-stimulated progesterone synthesis. In muscle cells, PLIN3 overexpression increases triacylglycerol accumulation,^57^ suggesting that PLIN3 may act as a regulator of intracellular triacylglycerol content by limiting lipase activity. Given that luteal lipid droplets primarily contain triacylglycerol,^5^ a similar effect in luteal cells could lead to reduced progesterone synthesis and increased lipid droplet size. PLIN3 facilitates the transport of lysosomal hydrolases from the Golgi to lysosomes via mannose 6-phosphate receptors,^58^ which hydrolyze lysosomal cholesteryl esters from lipoproteins.^59^ This mechanism might explain the decreased progesterone synthesis with PLIN3 overexpression, possibly due to altered lysosomal lipid transfer to droplets. Further studies are needed to understand the function of PLIN3 in bovine luteal cells and steroidogenesis.

The luteolytic hormone, PGF2α, induces luteal regression in the bovine.^60^ Loss of progesterone and accumulation of lipid droplets in the cytoplasm of luteal cells is a characteristic of luteal regression reported across various species, including primates,^61^ domestic animals,^28,62^ and rodents.^63^ Perilipins, particularly PLIN2, play a crucial role in lipid droplet formation and metabolism regulation.^64,65^ Our study found that administering PGF2α led to an increase in PLIN2 mRNA expression while simultaneously decreasing the levels of PLIN3, PLIN4, and PLIN5, indicating a role in lipid droplet formation through the upregulation of PLIN2. In 3 T3-L1 cells, PGF2α augments the formation of lipid droplets and triacylglycerol synthesis via DGAT,^66^ whereas in hepatocytes, it reduces lipid droplet accumulation and promotes autophagy and lysosomal aggregation.^67^ This suggests that the impact of PGF2α on lipid droplets varies across cell types and likely involves modulation of various signaling pathways.

Our study investigates the impact of luteolytic and luteotropic hormones, specifically LH and PGF2α, on the expression of PLIN2. The cellular interactions and signaling pathways associated with these hormones in relation to luteal function are complex. While we have shed light on the role of perilipins in steroidogenesis, the exact molecular mechanisms underlying their influence have not been fully elucidated. This gap in understanding limits our ability to make definitive assertions about their specific roles regarding luteal function. Furthermore, the effects of genetic manipulation of PLIN2 on the lipid droplet proteome have yet to be determined. Conducting a comparative analysis of other proteins associated with lipid droplets under conditions of knockdown/overexpression would provide a more comprehensive understanding of the unique roles of perilipins in bovine luteal cells. Another aspect yet to be explored is the long-term impact of altering PLIN2 on follicular cell differentiation, luteal function, and reproductive outcomes. While our findings indicate changes in progesterone synthesis, further studies are needed to determine the precise molecular mechanisms and their implications for ovarian health and fertility.

## 5 | CONCLUSION

Our findings offer valuable insights into the regulation, function, and significance of the lipid droplet-associated protein, PLIN2, in bovine ovarian cells. We confirm the presence of PLIN2 in both ovarian follicular and steroidogenic luteal cells, particularly noting elevated levels during the transition from the follicular to luteal phase. Additionally, our research strongly supports the pivotal role of PLIN2 in regulating progesterone synthesis within the bovine corpus luteum. Moreover, our in vivo experiments establish a correlation between luteolytic hormone administration and PLIN2 expression levels, highlighting its regulatory significance in luteal function. Overall, our findings highlight PLIN2 as a promising therapeutic target for modulating luteal function in bovine species.

## Supplementary Material

supplement figs

## Figures and Tables

**TABLE 1:** Characteristics of antibodies used for Western blotting and microscopy.

Antibody Name	Dilution ratio	Species specificity	Source	Supplier (distributor, town, country)	Cat. No.
PLIN 2	1:1000	Bovine	Guinea Pig pAB	Fitzgerald Industries International (Acton, MA, USA)	20R-AP002
PLIN 3	1:1000	Bovine	Guinea Pig pAB	Fitzgerald Industries International	20R-TP001
STAR	1:10000	Mouse	Rabbit pAB	Abcam (Cambridge, United Kingdom)	ab96637
CYP11A1	1:1000	Mouse	Rabbit mAB	Cell Signaling (Boston, MA, USA)	14 217
HSD3B	1:1000	Bovine	Mouse mAB	Thermo Fisher (Waltham, MA, USA)	MA1–46438
VIM	1:1000	Bovine	Rabbit pAB	Abcam	ab137321
HSL	1:1000	Mouse	Rabbit pAB	Cell Signaling	4107
TUBB	1:5000	Bovine	Mouse mAB	Sigma Life Science (St. Louis, Missouri, USA)	T4026
ACTB	1:5000	Bovine	Mouse mAB	Sigma Life Science	A5441
Lipi-Blue	1 μM			Dojindo Molecular (Rockville, MD, USA)	LD01
BODIPY 493/503	10 μM			Thermo Fisher	D3922
HRP-linked	1:10000	Anti-guinea pig		Jackson ImmunoResearch (West Grove, PA, USA)	106–035–003
HRP-linked	1:10000	Anti-rabbit		Jackson ImmunoResearch	111–035–003
HRP-linked	1:10000	Anti-mouse		Jackson ImmunoResearch	115–035–205
DyLight 405	1:500	Anti-mouse		Jackson ImmunoResearch	115–475–166

Abbreviations: CYP11A1, Cholesterol side-chain cleavage enzyme; HSD3B, 3beta-Hydroxysteroid dehydrogenase; HSL, Hormone Sensitive Lipase; PLIN2, Perilipin 2; PLIN3, Perilipin 3; STAR, Steroidogenic acute regulatory protein; VIM, Vimentin; Beta-tubulin (TUBB; loading control); Beta-actin (ACTB; loading control).

## Data Availability

Microarray data are available at the NCBI GEO repository (accession number GSE83524), and RNA-Seq data are available at the NCBI GEO repository (accession number GSE217053). All other data will be provided at reasonable request.

## Abbreviations:

ACTB: beta actin
Ad: adenoviruses
βGal: beta-galactosidase
CE: cholesteryl esters
CYP11A1: cholesterol side-chain cleavage enzyme
dGC: differentiated granulosa cell
dTC: differentiated theca cell
FA: fatty acids
FSK: forskolin
GC: granulosa cell
HSD3B: 3-beta-hydroxysteroid dehydrogenase
HSL: hormone-sensitive lipase
LH: luteinizing hormone
LLC: large luteal cell
PBS: phosphate buffer solution
PGF2α: prostaglandin F2α
PKA: protein kinase A
PKC: protein kinase C
PLIN1: perilipin 1
PLIN2: perilipin 2
PLIN3: perilipin 3
PLIN4: perilipin 4
PLIN5: perilipin 5
PMA: phorbol myristate acetate
RIA: radioimmunoassay
siRNA: silencing RNA
SLC: small lueal cell
STAR: steroidogenic acute regulatory protein
TBS: tris-buffered saline
TC: theca cell
TG: triacylglycerol
VIM: vimentin

## References

[1] Olzmann JA, Carvalho P. Dynamics and functions of lipid droplets. Nat Rev Mol Cell Biol. 2019;20(3):137–155. 30523332 10.1038/s41580-018-0085-z PMC6746329

[2] Plewes MR, Talbott HA, Saviola AJ, Woods NT, Schott MB, Davis JS. Luteal lipid droplets: a novel platform for steroid synthesis. Endocrinology. 2023;164(9):bqad124. 37586092 10.1210/endocr/bqad124 PMC10445418

[3] Raviv S, Hantisteanu S, Sharon SM, Atzmon Y, Michaeli M, Shalom-Paz E. Lipid droplets in granulosa cells are correlated with reduced pregnancy rates. J Ovarian Res. 2020;13(1):1–10. 10.1186/s13048-019-0606-1 PMC6945749 31907049

[4] Itabe H, Yamaguchi T, Nimura S, Sasabe N. Perilipins: a diversity of intracellular lipid droplet proteins. Lipids Health Dis. 2017;16(1):1–11. 28454542 10.1186/s12944-017-0473-y PMC5410086

[5] Talbott HA, Plewes MR, Krause C, Formation and characterization of lipid droplets of the bovine corpus luteum. Sci Rep. 2020;10(1):11287. 32647143 10.1038/s41598-020-68091-2 PMC7347867

[6] Xu G, Sztalryd C, Lu X, Post-translational regulation of adipose differentiation-related protein by the ubiquitin/proteasome pathway. J Biol Chem. 2005;280(52):42841–42847. 16115879 10.1074/jbc.M506569200

[7] Gao S, Gan X, He H, Dynamic characteristics of lipid metabolism in cultured granulosa cells from geese follicles at different developmental stages. Biosci Rep. 2019;39(12):BSR20192188. 31808518 10.1042/BSR20192188 PMC6928526

[8] Khan R, Jiang X, Hameed U, Shi Q. Role of lipid metabolism and signaling in mammalian oocyte maturation, quality, and acquisition of competence. Front Cell Dev Biol. 2021;9:639704. 33748128 10.3389/fcell.2021.639704 PMC7973101

[9] Ibayashi M, Aizawa R, Mitsui J, Tsukamoto S. Lipid droplet synthesis is associated with angiogenesis in mouse ovarian follicles. Biol Reprod. 2023;108(3):492–503. 36579469 10.1093/biolre/ioac223

[10] Plewes MR, Krause C, Talbott HA, Trafficking of cholesterol from lipid droplets to mitochondria in bovine luteal cells: acute control of progesterone synthesis. FASEB J. 2020;34(8):10731–10750. 32614098 10.1096/fj.202000671R PMC7868007

[11] Williams CJ, Erickson GF. Morphology and Physiology of the Ovary. Endotext; 2015.

[12] Filicori M The role of luteinizing hormone in folliculogenesis and ovulation induction. Fertil Steril. 1999;71(3):405–414. 10065772 10.1016/s0015-0282(98)00482-8

[13] Przygrodzka E, Plewes MR, Davis JS. Luteinizing hormone regulation of inter-organelle communication and fate of the corpus luteum. Int J Mol Sci. 2021;22(18):9972. 34576135 10.3390/ijms22189972 PMC8470545

[14] Kraemer FB, Shen W-J, Harada K, Hormone-sensitive lipase is required for high-density lipoprotein cholesteryl ester-supported adrenal steroidogenesis. Mol Endocrinol. 2004;18(3):549–557. 14657254 10.1210/me.2003-0179

[15] Rodgers R, O’Shea J, Findlay J. Progesterone production in vitro by small and large ovine luteal cells. Reproduction. 1983;69(1):113–124. 10.1530/jrf.0.0690113 6310106

[16] McCracken JA, Custer EE, Lamsa JC. Luteolysis: a neuroendocrine-mediated event. Physiol Rev. 1999;79(2):263–323. 10221982 10.1152/physrev.1999.79.2.263

[17] Bishop CV, Xu F, Steinbach R, Changes in immune cell distribution and their cytokine/chemokine production during regression of the rhesus macaque corpus luteum. Biol Reprod. 2017;96(6):1210–1220. 28575196 10.1093/biolre/iox052 PMC6279079

[18] Douglas R, Ginther O. Luteolysis following a single injection of prostaglandin F2α in sheep. J Anim Sci. 1973;37(4):990–993. 4795792 10.2527/jas1973.374990x

[19] Peterson A, Fairclough R, Payne E, Smith J. Hormonal changes around bovine luteolysis. Prostaglandins. 1975;10(6):675–684. 1239057 10.1016/s0090-6980(75)80015-3

[20] Estill CT, Britt JH, Gadsby JE. Repeated administration of prostaglandin F2α during the early luteal phase causes premature luteolysis in the pig. Biol Reprod. 1993;49(1):181–185. 8353186 10.1095/biolreprod49.1.181

[21] Silvia W The role of uterine and ovarian hormones in luteolysis: a comparison among species. Reprod Domest Anim. 1999;34(3–4):317–328.

[22] Behrman H, Luborsky-M oore J, Pang C, Wright K, Dorflinger L. Mechanisms of PGF 2α action in functional luteolysis. In: Channing CP, Marsh JM, Sadler WA, eds. Ovarian Follicular and Corpus Luteum Function. Springer; 1979:557–575. 223398

[23] Zhang X, Li J, Liu J, Luo H, Gou K, Cui S. Prostaglandin F2a upregulates slit/Robo expression in mouse corpus luteum during luteolysis. J Endocrinol. 2013;218(3):299–310. 23814012 10.1530/JOE-13-0088

[24] Antonini R, Turner TT, Pauerstein CJ. The hormonal control of the Guinea pig corpus luteum during early pregnancy. Fertil Steril. 1976;27(11):1322–1325. 976506 10.1016/s0015-0282(16)42203-x

[25] Koering MJ. Luteolysis in normal and prostaglandin F2α-treated pseudopregnant rabbits. Reproduction. 1974;40(2):259–267. 10.1530/jrf.0.0400259 4372343

[26] Kim SO, Markosyan N, Pepe GJ, Duffy DM. Estrogen promotes luteolysis by redistributing prostaglandin F2α receptors within primate luteal cells. Reproduction. 2015;149(5):453–464. 25687410 10.1530/REP-14-0412 PMC4380810

[27] Waterman R Changes in lipid contents and fatty acid compositions in ovine corpora lutea during the estrous cycle and early Pregnan. Biol Reprod. 1988;38(3):605–615. 3378073 10.1095/biolreprod38.3.605

[28] Talbott H, Davis JS. Lipid droplets and metabolic pathways regulate steroidogenesis in the corpus luteum. Life Cycle Corpus Luteum. 2017;1:57–78.

[29] Khanthusaeng V, Thammasiri J, Bass CS, Lipid droplets in cultured luteal cells in non-pregnant sheep fed different planes of nutrition. Acta Histochem. 2016;118(6):553–559. 27388430 10.1016/j.acthis.2016.05.007

[30] Sawyer H Structural and functional properties of the corpus luteum of pregnancy. J Reprod Fertil. 1995;97:110. 7623352

[31] Summers AF, Pohlmeier WE, Sargent KM, Altered theca and cumulus oocyte complex gene expression, follicular arrest and reduced fertility in cows with dominant follicle follicular fluid androgen excess. PLoS One. 2014;9(10). 10.1371/journal.pone.0110683 PMC4199720 25330369

[32] Ireland JJ, Murphee R, Coulson P. Accuracy of predicting stages of bovine estrous cycle by gross appearance of the corpus luteum. J Dairy Sci. 1980;63(1):155–160. 7372895 10.3168/jds.S0022-0302(80)82901-8

[33] Mao D, Hou X, Talbott H, Cushman R, Cupp A, Davis JS. ATF3 expression in the corpus luteum: possible role in luteal regression. Mol Endocrinol. 2013;27(12):2066–2079. 24196350 10.1210/me.2013-1274 PMC3857195

[34] Hansel W, Alila HW, Dowd JP, Yang X. Control of steroidogenesis in small and large bovine luteal. Cells Aust J Biol Sci. 1987;40(3):331–348. 3327492 10.1071/bi9870331

[35] Weber DM, Fields PA, Romrell LJ, Functional differences between small and large luteal cells of the late-pregnant vs. nonpregnant cow. Biol Reprod. 1987;37(3):685–697. 3676412 10.1095/biolreprod37.3.685

[36] Romereim SM, Summers AF, Pohlmeier WE, Transcriptomes of bovine ovarian follicular and luteal cells. Data Brief. 2017;10:335–339. 28004024 10.1016/j.dib.2016.11.093 PMC5157705

[37] Romereim SM, Summers AF, Pohlmeier WE, Gene expression profiling of bovine ovarian follicular and luteal cells provides insight into cellular identities and functions. Mol Cell Endocrinol. 2017;439:379–394. 27693538 10.1016/j.mce.2016.09.029 PMC6711749

[38] Roy L, McDonald CA, Jiang C, Convergence of 3′, 5′-cyclic adenosine 5′-monophosphate/protein kinase A and glycogen synthase kinase-3β/β-catenin signaling in corpus luteum progesterone synthesis. Endocrinology. 2009;150(11):5036–5045. 19819952 10.1210/en.2009-0771 PMC3213761

[39] Li D, Yin X, Zmuda EJ, The repression of IRS2 gene by ATF3, a stress-inducible gene, contributes to pancreatic β-cell apoptosis. Diabetes. 2008;57(3):635–644. 18057093 10.2337/db07-0717

[40] Taurin S, Sandbo N, Yau DM, Sethakorn N, Dulin NO. Phosphorylation of β-catenin by PKA promotes ATP-induced proliferation of vascular smooth muscle cells. Am J Physiol Cell Physiol. 2008;294(5):C1169–C1174. 18353896 10.1152/ajpcell.00096.2008 PMC3327159

[41] Plewes MR, Hou X, Talbott HA, Luteinizing hormone regulates the phosphorylation and localization of the mitochondrial effector dynamin-related protein-1 (DRP1) and steroidogenesis in the bovine corpus luteum. FASEB J. 2020;34(4):5299–5316. 32077149 10.1096/fj.201902958R PMC7136153

[42] Youngquist R, Garverick H, Keisler D. Use of umbilical cord clamps for ovariectomy in cows. J Am Vet Med Assoc. 1995;207:474–475. 7591949

[43] Talbott H, Hou X, Qiu F, Early transcriptome responses of the bovine midcycle corpus luteum to prostaglandin F2α includes cytokine signaling. Mol Cell Endocrinol. 2017;452:93–109. 28549990 10.1016/j.mce.2017.05.018 PMC7388008

[44] Nafziger SR, Tenley SC, Summers AF, Attainment and maintenance of pubertal cyclicity may predict reproductive longevity in beef heifers. Biol Reprod. 2021;104(6):1360–1372. 33709137 10.1093/biolre/ioab044 PMC9630398

[45] Monaco CF, Plewes MR, Przygrodzka E, Basic fibroblast growth factor (FGF2) induces proliferation and collagen production by fibroblasts derived from the bovine corpus luteum. Biol Reprod. 2023;109(3):367–380. 37283496 10.1093/biolre/ioad065 PMC10502575

[46] Donaldson L, Hansel W. Histological study of bovine corpora Lutea1. J Dairy Sci. 1965;48(7):905–909. 14330748 10.3168/jds.s0022-0302(65)88360-6

[47] Duffy DM, Ko C, Jo M, Brannstrom M, Curry TE Jr. Ovulation: parallels with inflammatory processes. Endocr Rev. 2019;40(2):369–416. 30496379 10.1210/er.2018-00075 PMC6405411

[48] Sztalryd C, Brasaemle DL. The perilipin family of lipid droplet proteins: gatekeepers of intracellular lipolysis. Biochim Biophys Acta Mol Cell Biol Lipids. 2017;1862(10):1221–1232. 28754637 10.1016/j.bbalip.2017.07.009 PMC5595658

[49] Rowe ER, Mimmack ML, Barbosa AD, Conserved amphipathic helices mediate lipid droplet targeting of perilipins 1–3. J Biol Chem. 2016;291(13):6664–6678. 26742848 10.1074/jbc.M115.691048 PMC4807253

[50] Shen WJ, Azhar S, Kraemer FB. Lipid droplets and steroidogenic cells. Exp Cell Res. 2016;340(2):209–214. 26639173 10.1016/j.yexcr.2015.11.024 PMC4744538

[51] Li Y, Khanal P, Norheim F, Plin2 deletion increases cholesteryl ester lipid droplet content and disturbs cholesterol balance in adrenal cortex. J Lipid Res. 2021;62:100048. 33582145 10.1016/j.jlr.2021.100048 PMC8044703

[52] Ueno M, Suzuki J, Hirose M, Cardiac overexpression of perilipin 2 induces dynamic steatosis: prevention by hormone-sensitive lipase. Am J Physiol Endocrinol Metab. 2017;313(6):E699–E709. 28851734 10.1152/ajpendo.00098.2017 PMC6415650

[53] Imamura M, Inoguchi T, Ikuyama S, ADRP stimulates lipid accumulation and lipid droplet formation in murine fibroblasts. Am J Physiol Endocrinol Metab. 2002;283(4):E775–E783. 12217895 10.1152/ajpendo.00040.2002

[54] Listenberger LL, Ostermeyer-Fay AG, Goldberg EB, Brown WJ, Brown DA. Adipocyte differentiation-r elated protein reduces the lipid droplet association of adipose triglyceride lipase and slows triacylglycerol turnovers. J Lipid Res. 2007;48(12):2751–2761. 17872589 10.1194/jlr.M700359-JLR200

[55] Lu X, Gruia-Gray J, Copeland NG, The murine perilipin gene: the lipid droplet-associated perilipins derive from tissue-specific, mRNA splice variants and define a gene family of ancient origin. Mamm Genome. 2001;12(9):741–749. 11641724 10.1007/s00335-01-2055-5

[56] Nose F, Yamaguchi T, Kato R, Crucial role of perilipin-3 (TIP47) in formation of lipid droplets and PGE2 production in HL-60-derived neutrophils. PLoS One. 2013;8(8):e71542. 23936516 10.1371/journal.pone.0071542 PMC3731282

[57] Kleinert M, Parker BL, Chaudhuri R, mTORC2 and AMPK differentially regulate muscle triglyceride content via Perilipin 3. Mol Metabol. 2016;5(8):646–655. 10.1016/j.molmet.2016.06.007 PMC5021677 27656402

[58] Díaz E, Pfeffer SR. TIP47: a cargo selection device for mannose 6-phosphate receptor trafficking. Cell. 1998;93(3):433–443. 9590177 10.1016/s0092-8674(00)81171-x

[59] Dubland JA, Francis GA. Lysosomal acid lipase: at the crossroads of normal and atherogenic cholesterol metabolism. Front Cell Dev Biol. 2015;3:3. 25699256 10.3389/fcell.2015.00003 PMC4313778

[60] Pate J, Hughes C. Luteal prostaglandins: mechanisms regulating luteal survival and demise in ruminants. Animal. 2023;17:100739. 37567666 10.1016/j.animal.2023.100739

[61] Fraser H, Lunn S, Harrison D, Kerr JB. Luteal regression in the primate: different forms of cell death during naturaland gonadotropin-releasing hormone antagonist or prostaglandin analogue-induced luteolysis. Biol Reprod. 1999;61(6):1468–1479. 10569991 10.1095/biolreprod61.6.1468

[62] Deane HW, Hay MF, Moor R, Rowson L, Short R. The corpus luteum of the sheep: relationships between morphology and function during the oestrous cycle. Eur J Endocrinol. 1966;51(2):245–263. 10.1530/acta.0.0510245 4951866

[63] Strauss JF 3rd, Seifter E, Lien EL, Goodman DB, Stambaugh RL. Lipid metabolism in regressing rat corpora lutea of pregnancy. J Lipid Res. 1977;18(2):246–258. 845506

[64] Heid HW, Moll R, Schwetlick I, Rackwitz H-R, Keenan TW. Adipophilin is a specific marker of lipid accumulation in diverse cell types and diseases. Cell Tissue Res. 1998;294:309–321. 9799447 10.1007/s004410051181

[65] Jiang H-P, Isolation Serrero G. and characterization of a full-length cDNA coding for an adipose differentiation-related protein. Proc Natl Acad Sci. 1992;89(17):7856–7860. 1518805 10.1073/pnas.89.17.7856 PMC49813

[66] Yamamoto Y, Taniguchi T, Inazumi T, Effects of the selective EP2 receptor agonist omidenepag on adipocyte differentiation in 3T3-L1 cells. J Ocular Pharmacol Therap. 2020;36(3):162–169. 10.1089/jop.2019.0079 PMC7175626 31934812

[67] Tian J, Du Y, Yu E, Prostaglandin 2α promotes autophagy and mitochondrial energy production in fish hepatocytes. Cells. 2022;11(12):1870. 35740999 10.3390/cells11121870 PMC9220818

