# Supplementary material for: Unraveling the role of lipid droplets and perilipin 2 in bovine luteal cells
Source: FASEB J. Author manuscript; Available in PMC 2024 Aug 26. (PMC11347014; doi:10.1096/fj.202400260RR)
Supplement: supplement figs [file NIHMS2016081-supplement-supplement_figs.pdf]

RIII Supporting Information Figures

Title: Unraveling the Role of Lipid Droplets and Perilipin 2 in Bovine Luteal Cells[1]

Running Title: Perilipin 2 in Bovine Luteal Cells

Key Words: Lipid Droplets, Perilipin 2, Steroidogenesis, Progesterone, Corpus Luteum, Prostaglandin F2α

Authors: Michele R. Plewes[2,3,4,#], Heather A. Talbott[2], Micah B. Schott[4], Jennifer R. Wood[5], Andrea S. Cupp[5], and John S. Davis[2,3,4]

[2]Olson Center for Women's Health, Department of Obstetrics and Gynecology, University of Nebraska Medical Center, 983255 Nebraska Medical Center, Omaha, NE 68198-3255, USA

[3]Veterans Affairs Nebraska Western Iowa Health Care System, 4101 Woolworth Ave, Omaha, NE 68105, USA

[4]Department of Biochemistry and Molecular Biology, University of Nebraska Medical Center, 985870 Nebraska Medical Center, Omaha, NE 68198-5870, USA

[5]Department of Animal Sciences, University of Nebraska–Lincoln, Lincoln, NE 68583-0908, USA

[1]This project was supported by Agriculture and Food Research Initiative Competitive Grant no. 2018-67012-29531 (MRP) and 2017-67015-26450 (JSD) from the USDA National Institute of Food and Agriculture; IK2 BX004911-01A1 (MRP) and I01 BX004272 (JSD) from the U.S Department of Veterans Affairs; NIH grants R01 HD087402 and R01HD092263; and The Olson Center for Women’s Health. JSD is the recipient of a VA Senior Research Career Scientist Award IK6 BX005797.

[#]**Correspondence:** Michele R. Plewes, Olson Center for Women's Health, University of Nebraska Medical Center, 983255 Nebraska Medical Center, Omaha, NE 68198-3255, USA.

E-mail: michele.plewes@unmc.edu; ORCID: 0000-0002-6086-0104**;** Phone: 402-559-8627

Disclosure Statement: The authors have nothing to disclose.

Supporting Information Figure 1: PLIN2 is highly enriched in bovine luteal cells

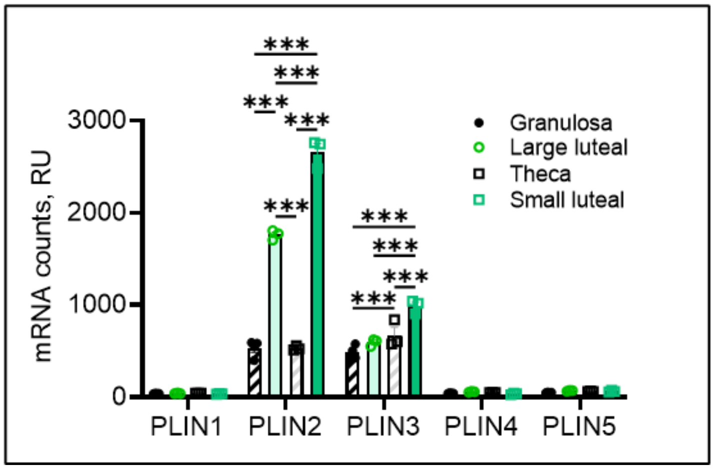

Supporting Information Figure 2: Lipid droplet accumulation increases as follicular cells undergoing the follicular to luteal transition.

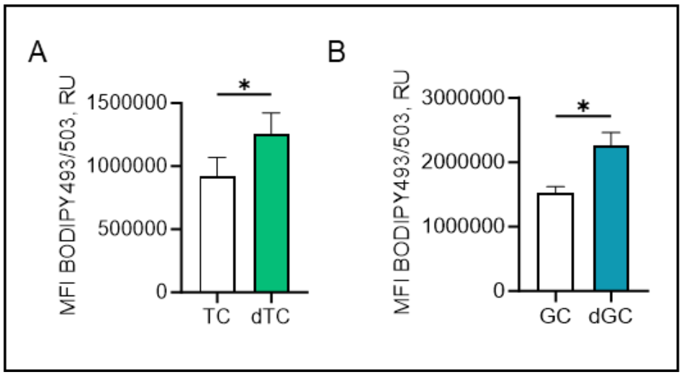

Revised Supporting Information Figure 3: Overexpression of Perilipin 3 (PLIN3) increases lipid droplet volume and attenuates progesterone production in bovine small luteal cells.

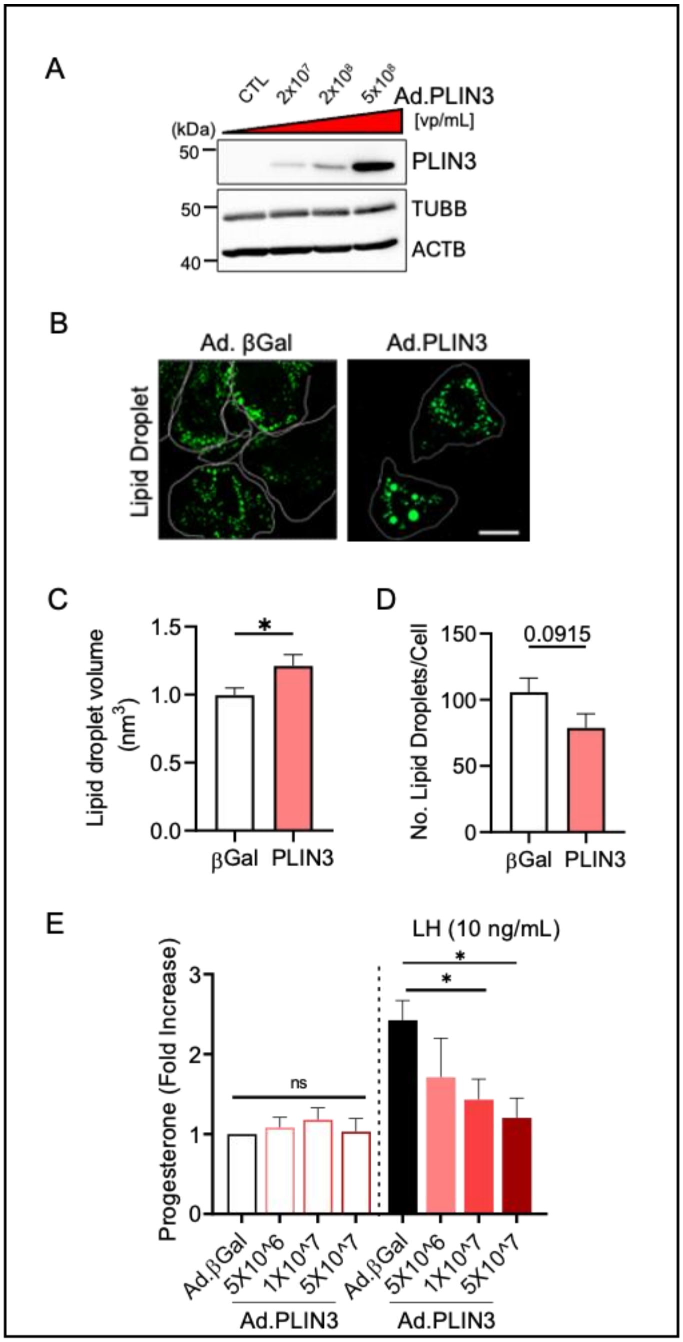

Supporting Information Figure 4: Effects of PGF2alpha on Perilipin mRNA expression *in vivo*.

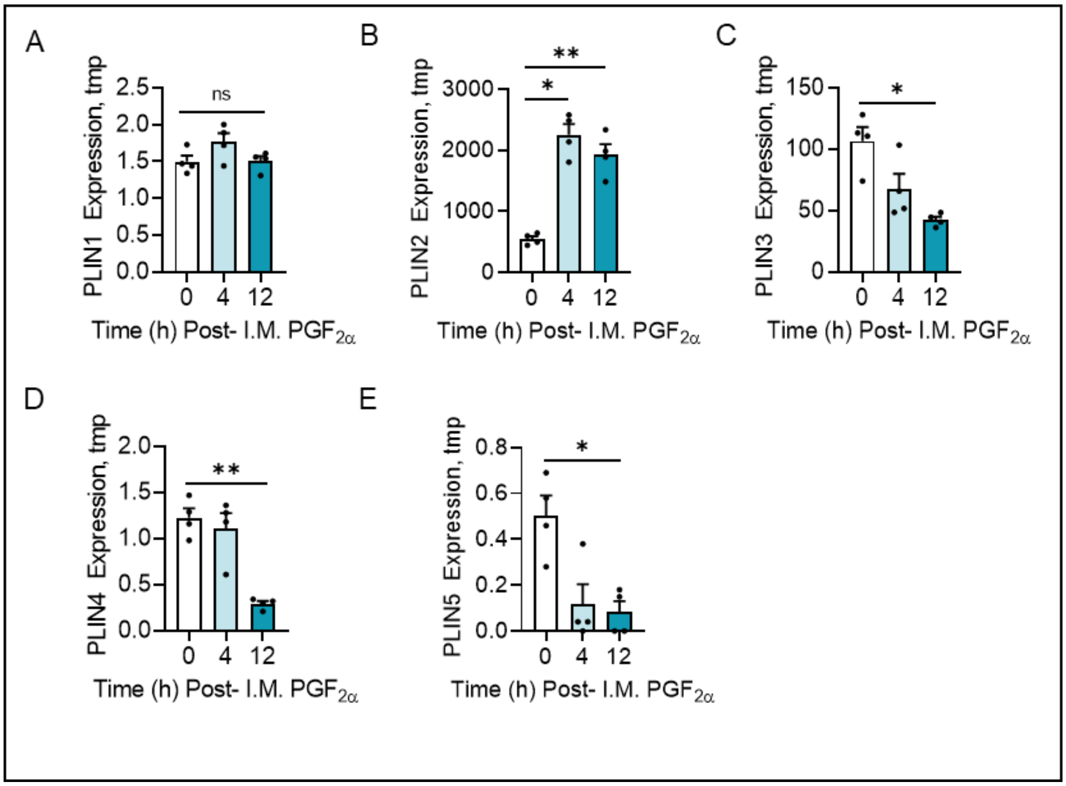

Supporting information Figure Captions

Supporting Information Figure 1: Microarray expression of Bovine Perilipins (1-5). We mined bovine gene expression arrays from the NCBI GEO repository (GSE83524) to analyze the expression of Perilipins (1-5) in freshly isolated bovine granulosa and theca cells from large follicles and purified preparations of bovine small and large luteal cells from mature corpora lutea. Microarray analysis of the Perilipin (1-5) family. Bovine granulosa cells (n = 4; open black hatch bars; circle symbol), theca cells (n = 3; open grey hatch bars; square symbol), large luteal cells (n = 3; closed light green bars; circle symbol), and small luteal cells (n = 3; closed green bars; square symbol) Significant differences were identified as changes greater than 1.5-fold and $P < 0.05$ supported by unpaired t-tests.

Supporting Information Figure 2: Lipid droplet accumulation increases as follicular cells undergoing the follicular to luteal transition. Bovine granulosa (GC) and theca cells (TC) were cultured for up to four days in medium contain 1% fetal calf serum with or without insulin/transferrin/selenium, the adenylyl cyclase activator forskolin (10 μM) and phorbol myristate acetate (PMA, 20 nM). (A) Quantitative analyses of the mean fluorescence intensity of BODIPY493/503 in TC or differentiated TC (dTC). (B) Quantitative analyses of the mean fluorescence intensity of BODIPY493/503 in GC or differentiated GC (dGC). Statistics were performed by *t*-tests to evaluate paired responses. Bars represent means ± sem. Significant difference between treatments, $*P < 0.05$.

Supporting Information Figure 3: Overexpression and knockdown of lipid droplet-associated proteins, Perilipin 3 (PLIN3), in bovine luteal cells. Replication-deficient adenoviruses (Ad) containing Beta-galactose (Ad.βGal; control) or PLIN3 (Ad.PLIN3) were utilized to overexpress PLIN3 in bovine small luteal cells. (A) Representative western blot of dose-dependent overexpression of Ad.PLIN3 in small luteal cells. Small luteal cells were infected with Ad.βGal or Ad.PLIN3 as described above. After 48 h, luteal cells were equilibrated for two hours and stimulated with luteinizing hormone (LH; 10 ng/mL) for 4 h. Small luteal cells were infected with Ad.βGal or Ad.PLIN3 and lipid droplets were labeled (Lipi-blue 1 μM) and visualized by confocal microscopy. (B) Representative micrographs of lipid droplets obtained from small luteal cells infected with Ad.βGal or Ad.PLIN3. (C) Quantification of lipid droplet volume (nm3) in small luteal cells infected with Ad.βGal or Ad.PLIN3. (D) Quantification of lipid droplet number and in small luteal cells infected with Ad.βGal or Ad.PLIN3. Statistics were performed by *t*-tests to evaluate paired responses. Data are means ± standard error, n=3. (E) Medium progesterone obtained from small luteal cells infected with Ad.βGal or increasing concentrations Ad.PLIN3 following stimulation with LH. Statistics were performed by two-way ANOVA was used to evaluate repeated measures with Tukey's multiple comparison tests. Bars represent means ± sem, n = 3. Significant difference between treatments, $*P < 0.05$. Micron bar represents 20 μm. Beta Tubulin (TUBB; loading control); Beta Actin (ACTB; loading control).

Supporting Information Figure 4: Effects of in vivo administration of Prostaglandin (PG) F2α on Perilipins (PLIN1-5) mRNA expression in bovine corpora lutea. Mid-luteal phase cows were injected with saline

(Control) or PGF2α, (25 mg, i.m.) and ovariectomized after 4- and 12-h to collect corpora lutea. RNA sequencing of whole luteal tissue was performed. (A) mRNA levels of PLIN1, (B) PLIN2, (C) PLIN3, (D) PLIN4, and (E) PLIN5 in the bovine corpus luteum at midcycle and 4- and 12 h post- PGF2α injection. Data are presented as mean number of transcripts per million (TPM) ± SEM. n = 4; *$p < 0.05$, **$p < 0.01$, compared to 0 h by DESeq2 analysis, Benjamini Hochberg correction. P-values shown are adjusted p-values for multiple comparisons.
